# Structural Insight into CVB3-VLP Non-Adjuvanted Vaccine

**DOI:** 10.3390/microorganisms8091287

**Published:** 2020-08-24

**Authors:** Minna M. Hankaniemi, Mo A. Baikoghli, Virginia M. Stone, Li Xing, Outi Väätäinen, Saana Soppela, Amirbabak Sioofy-Khojine, Niila V. V. Saarinen, Tingwei Ou, Brandon Anson, Heikki Hyöty, Varpu Marjomäki, Malin Flodström-Tullberg, R. Holland Cheng, Vesa P. Hytönen, Olli H. Laitinen

**Affiliations:** 1 Faculty of Medicine and Life Sciences, Tampere University, FI-33014 Tampere, Finland; outiva@outlook.com (O.V.); saana.soppela@tuni.fi (S.S.); amirbabak.sioofykhojine@tuni.fi (A.S.-K.); niila.saarinen@tuni.fi (N.V.V.S.); heikki.hyoty@tuni.fi (H.H.); olli.laitinen@tuni.fi (O.H.L.); 2 Department of Molecular and Cellular Biology, University of California, Davis, CA 95616, USA; mab@pioms.org (M.A.B.); lxing3@uci.edu (L.X.); tou@ucdavis.edu (T.O.); bjanson@ucdavis.edu (B.A.); rhch@ucdavis.edu (R.H.C.); 3 Institute for Molecular Medicine Finland (FIMM), HiLIFE, P.O. Box 20, University of Helsinki, 00014 Helsinki, Finland; 4 The Center for Infectious Medicine, Department of Medicine Huddinge, Karolinska Institutet, Karolinska University Hospital Huddinge, SE-141 52 Stockholm, Sweden; virginia.stone@ki.se (V.M.S.); malin.flodstrom-tullberg@ki.se (M.F.-T.); 5 Fimlab Laboratories, FI-33520 Tampere, Finland; 6 Department of Biological and Environmental Science/Nanoscience Center, University of Jyväskylä, P.O. Box 35, FI-40014 Jyväskylä, Finland; varpu.s.marjomaki@jyu.fi

**Keywords:** Coxsackievirus B (CVB), vaccine, virus-like particle (VLP)

## Abstract

Coxsackievirus B (CVB) enteroviruses are common pathogens that can cause acute and chronic myocarditis, dilated cardiomyopathy, aseptic meningitis, and they are hypothesized to be a causal factor in type 1 diabetes. The licensed enterovirus vaccines and those currently in clinical development are traditional inactivated or live attenuated vaccines. Even though these vaccines work well in the prevention of enterovirus diseases, new vaccine technologies, like virus-like particles (VLPs), can offer important advantages in the manufacturing and epitope engineering. We have previously produced VLPs for CVB3 and CVB1 in insect cells. Here, we describe the production of CVB3-VLPs with enhanced production yield and purity using an improved purification method consisting of tangential flow filtration and ion exchange chromatography, which is compatible with industrial scale production. We also resolved the CVB3-VLP structure by Cryo-Electron Microscopy imaging and single particle reconstruction. The VLP diameter is 30.9 nm on average, and it is similar to Coxsackievirus A VLPs and the expanded enterovirus cell-entry intermediate (the 135s particle), which is ~2 nm larger than the mature virion. High neutralizing and total IgG antibody levels, the latter being a predominantly Th2 type (IgG1) phenotype, were detected in C57BL/6J mice immunized with non-adjuvanted CVB3-VLP vaccine. The structural and immunogenic data presented here indicate the potential of this improved methodology to produce highly immunogenic enterovirus VLP-vaccines in the future.

## 1. Introduction

Coxsackievirus B3 (CVB3) is an RNA virus that belongs to the enterovirus genus of *Picornaviridae*. Infections caused by the six Coxsackievirus B serotypes (CVB1-6) usually manifest in mild flu-like symptoms or respiratory illness. However, CVBs can infect several organs after primary infection, such as the nervous system, pancreas, and heart [1]. Such disseminated infections can lead to severe organ damage and serious diseases including aseptic meningitis, encephalitis, pancreatitis, and myocarditis. If myocarditis becomes chronic, it can lead to dilated cardiomyopathy (DCM) which is a leading cause of heart transplantation. CVB3 is the enterovirus most often responsible for viral myocarditis [2] and it can also cause hand, foot, and mouth disease (HFMD) outbreaks [3]. CVBs have also been associated with the development of type 1 diabetes [4,5,6]. Therefore, CVBs have been considered as potent targets for the future enterovirus vaccines [6,7,8].

Besides polio [9] and Enterovirus-71 [10] (EV-A71) vaccines, no clinically approved treatments or vaccines are available against enteroviruses. To meet the demand for the prevention of CVB associated diseases, the clinical development of a polyvalent CVB vaccine has recently commenced [6]. CVB vaccines in clinical development and EV-vaccines in clinical use contain either live-attenuated viruses (oral polio vaccine, OPV) or formalin inactivated viruses (CVB vaccine, inactivated polio vaccine, EV-A71-vaccine). These vaccines have worked well in the prevention of enterovirus diseases. However, as a live vaccine, OPV faces safety issues, due to the reversion of the attenuated vaccine virus strain towards the virulent strain in the vaccine recipients leading to vaccine-associated paralytic polio and subsequent polio outbreaks [11]. Thus, the use of OPV is becoming difficult, however two of the three wild poliovirus strains have been declared to be eradicated. Inactivated poliovirus vaccine has been effective and safe, but this vaccine technology does not allow flexible epitope engineering and it cannot be used for enterovirus types which do not grow well in cell cultures. With this comes an increasing demand for new vaccine technologies and cost-effective vaccine production methods that allow flexible vaccine engineering and the large-scale production of vaccines against different enterovirus types.

Virus-like particles (VLPs) are empty particles assembled from recombinantly expressed viral structural proteins, which ensures therefore, that they are non-infectious and can be produced for viruses which do not grow well in cell cultures. Thus, VLP-based vaccines are not associated with the safety concerns related to live virus vaccines and offer also important advantages in the vaccine engineering and manufacturing process. Promising VLP-based vaccine candidates against EVs have been produced as demonstrated by monovalent VLP-vaccines against CVB3 [12,13], EV-A71 [14], CVA6 [15], CVA16 [16], and EV-D68 [17] or polyvalent VLP-vaccines against EV-A71, CVA6, CVA10, and CVA16 [18]. We have previously produced CVB3-VLPs with moderate yields (yield referring to the total amount of the pure end product protein) [13], while the efficient production of CVB1-VLPs was described based on a new construct design, use of a baculoviral (flashBAC) genome, and an improved purification method [19]. In the present study, we aimed to enhance CVB3-VLP production yields and purity as well as define an industrially scalable production process. Due to these aims, CVB3-VLPs were produced utilizing a production process similar to that described for CVB1-VLP [19].

Enteroviruses are non-enveloped icosahedral viruses that are around 30 nm in diameter. The capsid protects an approximately 7.5 kb single-stranded positive-sense RNA genome, which is translated into a polyprotein that is co-translationally and post-translationally cleaved by the viral proteases 2A and 3CD to yield structural proteins VP0, VP1, and VP3. These structural proteins assemble into the viral capsid, wherein the viral RNA is encapsidated. VP0 is further processed into VP2 and VP4 in a viral RNA-driven autocatalysis reaction yielding the mature virus [20]. The basic building block (protomer) of the icosahedral capsid contains VP1-VP4 proteins and the entire capsid consists of 60 such protomers [21]. In mature enteroviruses, VP4 together with the N-terminus of VP1 are located at the internal surface of the capsid [21]. On the outer surface of the capsid, there are three-fold propeller-like protrusions, star-shaped five-fold plateaus (called mesa), and depressions (called canyons) surrounding each plateau [21]. The canyon, often contains the enterovirus receptor-binding site and binding at the canyon results in a cascade of structural changes [22], ultimately leading to the release of the N-terminus of VP1 and VP4 [23] to form the expanded 135S cell-entry intermediate (also called A-particle) [24]. High-resolution structures of CVB-VLPs have thus far remained unavailable. As such, here, we aimed to determine the native structure of CVB3-VLPs by cryoEM and image reconstruction. Structural information provides the possibility to engineer both the stability of the particle and it’s immunogenic properties. We also compared the VLP structure resolved in this study to the published CVB3 and CVA16 whole-virus structures to identify possible differences between the mature virus and the VLP.

Our previously produced CVB3-VLP vaccine induced a robust neutralizing antibody response in mice, however this result was obtained by utilizing a strong Freund’s adjuvant [13] which is not allowed for human use. Therefore, we studied the immunogenicity of non-adjuvanted CVB3-VLP vaccine in mice.

## 2. Materials and Methods

### 2.1. Generation of the CVB3-VLP-Encoding Baculoviruses

The baculoviral transfer vector pOET5, containing separate cassettes for the CVB3 VP1-4 capsid region under the control of the polyhedrin promoter and the 3CD-protease under a CMV promoter control was ordered from GeneArt (Regensburg, Germany). CMV promoter acts as a weak promoter in insect cells, decreasing the toxic effects by the 3CD protease [25]. The fully sequenced CVB3 strain (GenBank accession number M33854.1) was used as a template. The recombinant baculovirus was produced according to the flashBAC baculovirus expression system manual utilizing the flashBAC ULTRA baculovirus genome. Baculovirus working stock titer was determined using BacPAK Baculovirus Rapid Titer Kit (Clontech, Mountain View, CA, USA).

### 2.2. Production, Purification and Chracterization of CVB3-VLPs

CVB3-VLPs were produced in High-Five insect cells with a multiplicity of infection (MOI) value of 5. The VLP containing production medium was clarified by mixing it with 20 g/L Sartoclear Dynamics Lab filter aid (diatomaceous earth) and then filtering it through a 0.2 µm filter. CVB3-VLPs were concentrated from the culture supernatant by tangential flow filtration (TFF), utilizing 750 MWCO hollow fiber with an ÄKTA Flux system. The buffer was exchanged to 40 mM Tris pH 7.3, 10 mM MgCl_2_, 40 mM NaCl, and 0.1% Tween80 with the same system, and the impurities were removed from the preparation using a HiLoad 26/10 Q Sepharose High Performance anion exchange column (GE Healthcare). The VLPs were captured into CIMmultus SO_3_ cation exchange chromatography column (BIASeparations) and were eluted using NaCl gradient (pure VLP eluted with ~250 mM NaCl).

Purified VLPs were analyzed in mini-PROTEAN TGX stain-free precast gradient gels (4–20%) (BioRad, Finland). VLP proteins and impurities were assessed by densitometry analysis of SDS-PAGE gels using the ImageJ software [26]. VP1 and VP3 proteins were detected by Western blotting using in-house produced rabbit anti-CVB1-6 polyclonal antibody [19] and IRDye-labeled secondary antibody. Determination of VLP total protein concentration and dynamic light scattering (DLS) analysis were performed as described [27]. The thermal stability of the VLP particle was characterized by differential scanning fluorimetry (DSF) as described in [19]. The VLP was also characterized by transmission electron microscopy (TEM). Formvar coated and carbon evaporated copper grids were made hydrophilic by UV-C irradiation at 254 nm (2.0 mWatts/cm^2^) for 10 min. Then, 5 µL of VLP (0.5 mg/mL) was added on the grid and incubated for 20 s, after which the excess VLP was removed by blotting with Whatman 3MM paper. The VLPs were negatively stained through the addition of 5 µL 1% Uranyl acetate on the grid for 20 s which was also removed by blotting. The grids were dried overnight and imaged with a F200 S/TEM (JEOL) transmission electron microscope.

### 2.3. Cryo-Electron Microscopy Structural Determination of CVB3-VLP

Data collection was performed using a JEOL JEM 2100F TEM operating at 200 kV. Briefly, a 3.5 µL of CVB3-VLP containing solution with final concentration of 1.2 μg/mL was placed on Quantifoil R2/2 Cu 300 mesh grids. After a 1.5-min incubation time, 0.5 µL of NP40 detergent was added for 15 s. The excess solution was removed by automated blotting and quickly plunged into liquid ethane. The frozen-hydrated CVB3-VLPs were transferred into the EM using a Gatan 626 cryo-transferring system. The specimen was examined at 80,000 magnification and images were captured using a CCD camera with a pixel size of 1.6 Ångstroms using SerialEM autofocusing software with defocus range set to 0.5–2.5 Ångstroms. Digital images with no stigmatism or drift were selected for further analysis.

Cryo-EM micrographs of CVB3-VLP were processed to generate a dataset for single particle analysis (SPA). Semi-automated particle selection [28] was employed to select a total of 5890 particles for refinement and reconstruction. Iterative de novo initial models were generated to determine and cross-validate the particle parameters. To optimize the 2D alignment and orientation determination during refinement, a polar Fourier transform (PFT) method [29] with radial range pushed to 10 Ångstroms beyond the particle edge was used in conjugation with a center search. The particle stack was further refined by Euler/center, defocus, scale-factor, and astigmatism screenings using JSPR. An iterative 3D refinement was carried out using the JSPR package [30]. To determine the final resolution of the 3D volume, the dataset was divided into two halves (even and odd numbered particles) [31,32]. Using eotest program from JSPR/EMAN packages [28,30], the final resolution of the CVB3-VLP was determined to be 10.2 Ångstroms based on the 0.5 Fourier shell correlation cut-off (data not shown).

### 2.4. Mouse Immunizations

Male C57BL/6J-mice (7–10 weeks old) were vaccinated by interscapular subcutaneous injections (i.s.) on days 0, 21, and 35 with 5 µg of non-adjuvanted CVB3-VLP vaccine (*n* = 5) or with vaccine buffer (*n* = 5). Experiments with C57BL/6J mice were conducted at Karolinska Institutet, Stockholm, Sweden in accordance with the NIH Principles of Laboratory Animal Care and national laws in Sweden and were approved by the local ethics committee (permission number S 48–14). Blood samples were collected prior to each immunization, on day 42 and at the experimental end point (day 49) from the tail vein or by terminal heart puncture (day 49) and used to isolate serum.

### 2.5. CVB3-VLP Specific ELISA and Neutralization Assay

Two-fold serial dilutions from 1:200 diluted sera of individual mice were analyzed in ELISA for the presence of CVB3-specific IgG, IgG1 and IgG2a antibodies (Invitrogen, Saint Louis, MO, USA). Then, 96-well maxisorp plates (Nunc, Fisher Scientific, Vantaa, Finland) were coated with 100 ng of CVB3-VLPs per well (room temperature, overnight) in PBS. After washing, the plates were blocked with 0.1% BSA in PBS and the serum samples diluted in PBS containing 2% NaCl, 1% BSA and 0.05% Tween20 were applied. Horseradish peroxidase (HRP)-conjugated goat anti-mouse IgG (Sigma-Aldrich, Saint Louis, MO, USA), IgG1 or IgG2a (Invitrogen, Saint Louis, MO, USA) and Sigma FAST OPD substrate (Sigma-Aldrich, Saint Louis, MO, USA) were used for the detection of total IgG or IgG subtype responses. Absorbance (optical density, OD) was measured at 490 nm in a microplate reader (Victor 1420, Perkin Elmer, Turku, Finland). The background signal (wells without serum) was subtracted from all OD readings in the plate. A sample was considered positive if the net absorbance value was above the cut-off value calculated as follows (control mice mean OD + 3 × SD). Serum titres below 200 were considered negative.

CVB3 neutralizing antibodies were measured as previously described [19]. According to previous studies, uninfected animals that have not been vaccinated occasionally show a neutralizing antibody titre of 1:4 but not beyond this titre [33].

## 3. Results

### 3.1. Optimized Construct Design, Production Yields and Purity for CVB3-VLP

Previously, we have produced CVB3-VLPs [13] and CVB1-VLPs [19] in insect cells. CVB3-VLPs have been earlier produced using the Bac-to-Bac baculovirus-insect cell expression system, where the P1 polyprotein was expressed under polh promoter and 3CD protease was expressed under p10 promoter [13]. Co-expression of CVB3 P1 and 3CD led to P1 polyprotein cleavage into VP0, VP1 and VP3 by 3CD and subsequent VLP assembly [13]. However, the production yields of the VLPs were moderate. Subsequently we produced CVB1-VLPs and to enhance the VLP yield, we constructed a CVB1-VLP construct, in which the P1 polyprotein expression was under the strong polh promoter and 3CD protease under the weaker CMV promoter [19]. In the new design, the 3CD protease was produced in low levels, which decreases the toxic effects of 3CD on the host cells and allows the baculovirus-infected insect cells to produce higher amounts of VLP compared to the previous design. Also, the baculoviral genome (flashBAC ultra) utilized with the CVB1-VLP production contains deletions in the non-essential baculoviral protease genes [34], which increases the cell stability and lowers the burden on the baculovirus-infected cells, allowing for a more efficient production of the target protein. Another improvement made which enhanced the CVB1-VLPs production yield involved the selection of the insect cell line. CVB3-VLPs were produced in *Spodoptera frugiperda* sf9 insect cells [13], whereas CVB1-VLPs were produced in *Trichoplusia Ni* High Five insect cells [19], that have been shown to out-perform sf9 cells for VLP production yields [35]. During the current study, we applied the same construct design, insect cell line and purification method for producing CVB3-VLPs as that which we have previously used to produce CVB1-VLPs [19].

**Figure 1:**
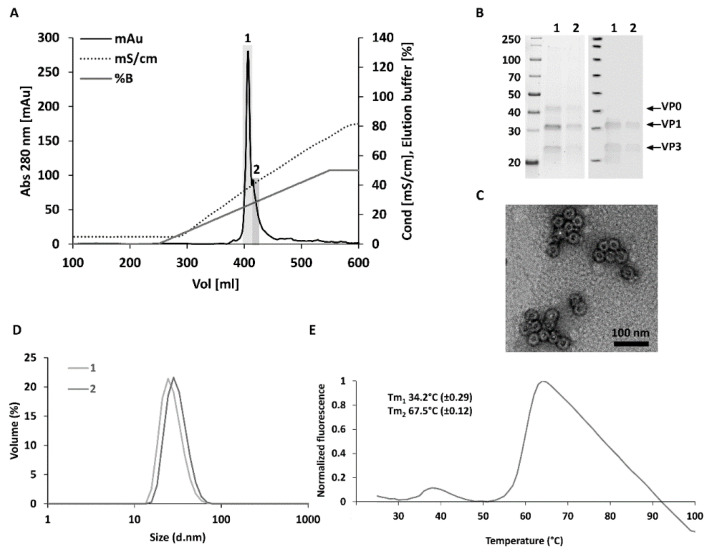
Purification and characterization of VLP. Final cation exchange chromatography concentration step and characterization of the purified CVB3-VLPs with SDS-PAGE, Western blotting, TEM, DLS and DSF. (**A**) In the final cation exchange chromatography step, the VLP was loaded on the column in a buffer containing 20 mM Tris pH 7.4, 5 mM MgCl_2_, 20 mM NaCl and 0.1% Tween80. Captured VLPs eluted in an approximately 250 mM NaCl concentration using a linear gradient into two fractions designated 1 and 2 (fraction 1 represented the most concentrated fraction and fraction 2 the more dilute side fraction; (**B**) SDS-PAGE and Western blot analyses of purified VLPs. The left panel shows the stainfree total protein staining of the purified CVB3-VLP fractions 1 and 2. The right panel shows VP1 and VP3 capsid protein detection by Western blot using an in-house produced rabbit anti-CVB1-6 polyclonal antibody; (**C**) Transmission Electron Microscopy (TEM) image of the chromatography purified CVB3-VLP. Scale bar 100 nm, 50,000× magnification. (**D**) Dynamic light scattering (DLS) analysis of the purified CVB3-VLP fractions. (**E**) Thermal stability profile of CVB3-VLP with differential scanning fluorimetry (DSF). Fluorescence intensity of the dye in the presence of VLPs was plotted as a function of the temperature, and melting temperatures (Tm) of the VLPs were derived from the inflection points of the transition curve using the Boltzmann equation [36]. The data is shown as an average of three independent measurements ± Standard Deviation.

CVB3-VLPs produced in High Five insect cells were extracted from the cell culture supernatants with tangential flow filtration and subsequently purified with anion and cation exchange chromatographic steps. First, the impurities were removed from the concentrated VLP with anion exchange chromatography. Then, CVB3-VLPs were captured into the SO_3_ cation exchange chromatography column and were eluted from the column with a linear NaCl gradient. The VLPs eluted from the column at a NaCl concentration of ~250 mM into two fractions (Figure 1A). SDS-PAGE analysis and the subsequent detection of proteins with a stain-free staining method (Figure 1B, left panel) revealed three prominent proteins of approximately 38 kDa, 31 kDa, and 26 kDa corresponding to the CVB3 capsid proteins VP0, VP1 and VP3. VP1 and VP3 capsid proteins were also detected with rabbit anti-CVB1-6 polyclonal antibody in Western Blot analysis (Figure 1B, right panel). According to TEM-analysis, the particles were intact and had the correct morphology and an average size of 30 nm (Figure 1C). DLS analysis showed, that the first and the second fraction contained 100% particles with average hydrodynamic diameters of 27.5 (±0.2) nm and 31.1 (±1.5) nm respectively and the samples were very monodisperse (polydispersity indexes, PdI: fraction 1: 0.11 ± 0.02, fraction 2: 0.24 ± 0.01) (Figure 1D). These sizes were comparable with the CVB1-VLPs [19] and CVB3-VLPs [13] we studied previously. Differential scanning fluorimetry (DSF) was used to analyze the thermal stability of CVB3-VLP. As we have shown previously for CVB1-VLP, the thermal stability of CVB3-VLP was lower than that of native viruses and comparable to the thermal stability of CVB1-VLP [19]. Two melting temperatures (Tm) were determined for CVB3-VLP, Tm_1_ (34.2 °C ± 0.29) that can be assigned to the initial capsid unfolding and expansion and Tm_2_ (67.5 °C ± 0.12) that can be assigned to the denaturation of the VLPs (Figure 1E).

### 3.2. Structural Analysis of CVB3-VLP

**Figure 2:**
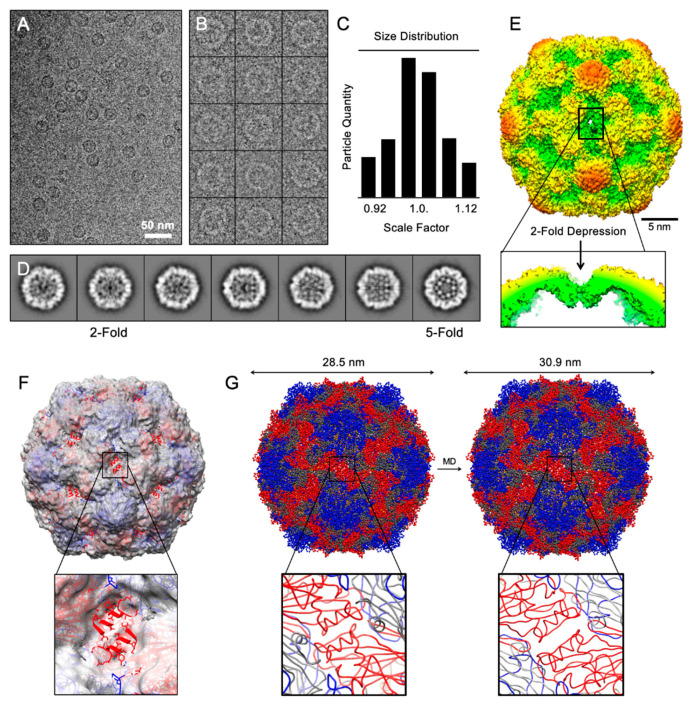
Structural analysis of CVB3 virus-like particle. (**A**) Field view of cryo-EM micrographs of frozen-hydrated CVB3-VLPs. (**B**) A gallery of selected particles used towards structure determination (inverted). (**C**) Particle sorting by scale factor using Polar Fourier Transform method. Particles within ±3% of the average were used to calculate 2D-referece-free class averages shown in (**D**). (**E**) 3D surface rendering 2-fold view of CVB3-VLP colored by radial distance from cold to warm. A depression at 2-fold axis is pointed out. (**F**,**G**) Rigid-body fitting and dynamic flexible fitting of CVA6-VLP into cryo-EM density revealing the expanded size as a result of 2-fold depression.

High-resolution structural information is available for several enteroviruses such as CVB3 [21]. However, the structure of CVB-VLPs has remained unavailable. Cryo-EM images of CVB3-VLP revealed spherical particles of uniform size with on average, a diameter of ~31 nm (cryo-EM field view in Figure 2A and contrast inverted selected particles in Figure 2B). The overall three-dimensional structure of CVB3-VLPs (resolved here by cryo-EM single particle analysis) is similar to CVA6-VLP, EV-A71-VLP, and CVA16 135s particles and is ~2 nm larger than the mature virion of EV-A71 and CVA16, as well as the CVB3 X-ray structure [37,38,39,40,41]. Using a cross-correlative circular averaging technique introduced by Cheng et al. in 1996 [29,31], the icosahedral particles were sorted by a scale factor based on statistical measures (Figure 2C). To ensure a homogeneous population for single particle analysis, particles with a diameter larger than ~34 nm and smaller than ~27 nm were withdrawn from the analysis; these particles were generally broken and/or collapsed, with irregular shape and size. The remaining set of images were further sorted based on Euler orientations (θ, ω, Φ) into two-dimensional class averages (Figure 2D), from which an iterative de nova initial model generation was carried out. The resolved CVB3-VLP cryo-EM structure has been deposited into worldwide protein data bank with ID: EMD-22495.

From the cryo-EM density map, the mesa (star-shaped plateau at five-fold axis), the canyon surrounding the five-fold axis, and propeller-like features at the three-fold axis are clearly resolved (Figure 2E). Rigid-body fitting of EV-A71 (PDB: 3VBF) revealed that the two adjacent alpha helices from VP0 do not fit into the density map. A depression at the two-fold icosahedral axis is observed, which illustrates an open conformation at the junction channel and is similar to CVA6-VLP, EV-A71-VLP, and CVA16 135s particles. After detecting the above mentioned similarity of CVB3-VLP to CVA16 135s particles, we further characterized the differences between CVA16 mature virion and the CVB3-VLPs by carrying out computer simulations for molecular dynamic flexible fitting of atomic structures (using iMODfit [42] and PDB 5C4W [40]) to calculate the expansion of the CVB3-VLPs. We observed that the CVB3-VLP diameter is about 30.9 nm on average, in contrast to 28.5 nm of the CVA16 mature virion, an 8.4 % increase in the overall diameter (Figure 2F–G).

### 3.3. Non-Adjuvanted CVB3-VLPs Induce CVB3-Specific IgG, IgG1-, IgG2a and Neutralizing Antibody Responses in C57BL/6J Mice

**Figure 3:**
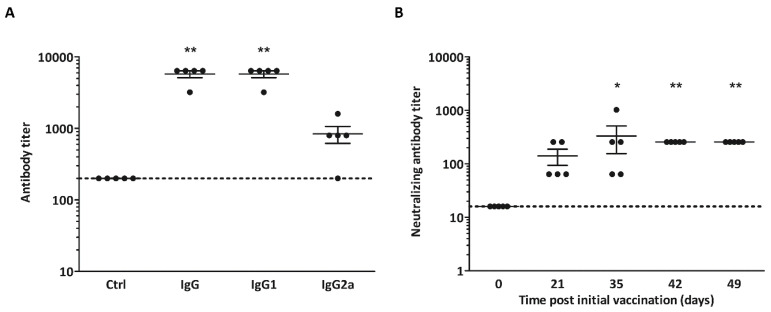
Immunization of mice with VLP. CVB3-VLP vaccines induced CVB3-specific serum IgG, IgG1-, IgG2a and neutralizing antibody responses in C57BL/6J-mice. (**A**) IgG, IgG1- and IgG2a ELISA at day 49 post initial vaccination. Antibody titers below 200 were assigned as negative (indicated by horizontal line); (**B**) CVB3 neutralizing antibody titers in the sera of mice immunized with CVB3-VLP vaccines in samples taken at day 0, 21, 35, 42 and 49 (*n* = 5). Mean IgG-, IgG1-, IgG2a- and neutralizing antibody titers are indicated by the line ±SEM. The dotted line represents the positive/negative threshold of neutralizing capacity (dilution 1:16) in the virus neutralization assay. * *p* < 0.05 and ** *p* < 0.01 compared to control mice or day 0 as determined by non-parametric Kruskal-Wallis test.

The immunogenicity of the CVB3-VLP vaccine was tested in five C57Bl/6J-mice by immunizing the animals on three occasions (days 0, 21 and 35) with 5 µg non-adjuvanted vaccine by i.s. injection. Serum samples were collected on days 0, 21, 35, 42, and 49. Control mice (*n* = 5) received vaccine buffer alone. Sera of C57BL/6J-mice immunized with CVB3-VLP vaccines or the vaccine buffer were analyzed for CVB3-specific serum IgG, IgG1 and IgG2a 49 days after the prime vaccination. All mice were positive for CVB3-specific IgG by ELISA with geometric mean titer (GMT) 5572 (Figure 3A). CVB3-specific IgG1 and IgG2a immunoglobulin subtypes which are indicators of Th2 and Th1 type immune responses were also studied. CVB3-VLP vaccine primarily induced primarily a Th2-type (IgG1) oriented response with GMT 5572, whereas the Th1-type (IgG2a) GMT was 696 in the sera from vaccinated animals (Figure 3A).

Sera from CVB3-VLP immunized mice were also analyzed for CVB3 neutralizing ability in vitro (Figure 3B). All vaccinated mice generated neutralizing antibodies by day 21 (after one vaccination) with GMT 111. After three vaccinations, the neutralizing GMT increased to 256 (*p* < 0.01).

## 4. Discussion

CVBs have constantly been among the top 15 enteroviruses most commonly reported to the CDC by diagnostic laboratories in the US that cause significant morbidity. Currently there are no treatments or vaccines available against CVBs, but the clinical development of a polyvalent formalin-inactivated whole-virus CVB vaccine is in progress [6,8]. Thus, there is a clear need to develop new vaccine technologies against CVBs. In addition to the traditional inactivated vaccine technologies, new VLP technologies can offer solutions for cost-effective manufacturing and epitope engineering of future CVB vaccines. Previously, we have produced CVB3-VLP [13] and CVB1-VLP [19] vaccines in insect cells and purified those with ion exchange chromatography. In the present study, our aim was to enhance the CVB3-VLP yield by utilizing a similar expression construct design and purification method to that employed for the CVB1-VLP [19]. As with the production of CVB1-VLP, here, we utilized a baculoviral (flashBAC) genome containing deletions in the non-essential protease genes [34] and the 3CD protease was produced in low levels by utilizing a CMV promoter. These changes reduce the toxic effects of baculoviral and [34] enteroviral [20] proteases on the production cell line. In the CVB3-VLP concentration step, the PEG-precipitation method used previously was replaced with tangential flow filtration and the impurities were removed from the concentrated VLP by anion exchange chromatography. The final cation exchange chromatographic capture and concentration step was performed as we have described previously [13,19]. The optimizations in the construct design, baculoviral genome, and purification method accounted for the enhanced production yield and purity of CVB3-VLP, and furthermore, this purification method is also a process that is compatible with industrial scale production. Since the construct design, production cell line and purification method utilized here differ to those used for the previously produced CVB3-VLP [13], it is impossible to address how much each change in the production process improved the final yield of the pure CVB3-VLP. However, we reached our objectives. Firstly, the developed purification method is scalable and utilize techniques generally used in industrial settings. Secondly, based on the characterization of the pure CVB3-VLP and the polydispersity Index (PdI) determined by DLS, both pure CVB3-VLP fractions were very monodisperse, demonstrating that compared to the previously developed purification method [13], CVB3-VLPs that were more homogenous were successfully purified. Thirdly, the obtained yield after purification was approximately 1.5 mg/L, which is three times higher than the yield of CVB3-VLP in our previous study [13].

An atomic-resolution structure of CVB3 virus was resolved by X-ray crystallography 25 years ago [21], however, similar structures of other CVBs are lacking. Also, the structures of CVB-VLPs have been unresolved until now. In the present study we determined a high-resolution cryo-EM three-dimensional structure of CVB3-VLP. The capsid surface structures characteristic for enteroviruses, i.e., mesa (star-shaped plateau at five-fold axis), the canyon surrounding the five-fold axis, and propeller-like features at the three-fold axis were clearly resolved from the VLP structure. According to the structure, CVB3-VLPs are ~2 nm larger than the mature virion of CVB3 [21] and the size difference is also similar to that seen when comparing EV-A71 and CVA16 viruses with their respective VLPs [37,38,39,40,41]. We found that the CVB3-VLP diameter is about 30.9 nm on average, which is very similar to the size of CVA6-VLP, EV-A71-VLP, and CVA16 135s particle (the expanded cell-entry intermediate, also called A-particle) [37,39,41]. The structure of CVB3-VLP axis closely resembles that of the expanded enterovirus A-particle. The enlargement of the two-fold axis is associated with mature virion-to-uncoating intermediate states [38]. The fenestration of viral A-particles near the two-fold axis has been shown for several native enteroviruses including Echovirus 1 and EV-A71 [43,44]. We saw a similar folding in the CVB3-VLP. In the A-particle, VP4 and the RNA-genome are still in the capsid and the particle is primed for infecting cells. Once the VP4 protein and the genome have been expulsed, the resulting empty capsid is referred to as empty particle (80S) [43]. Heating of the native enterovirus capsid results in the formation of both A- and empty-particles [43]. However, in physiological conditions enterovirus particles resemble the A-particles [44,45]. Since the structure of CVB3-VLP resembles that of A-particle, the VLP may closely resemble the form of mature virion in physiological conditions. Poliovirus capsid structure have been stabilized by heat selected mutants [46] and such mutations have been applied to generate poliovirus VLPs with better immunogenicity [47]. The importance of the solved CVB3-VLP structure is speeding up rational engineering of the CVB particle to be more stable by introducing point mutations and thus improving its immunogenicity similarly to poliovirus VLPs.

Most licensed VLP-vaccines contain adjuvants in their vaccine formulas [48] and to the best of our knowledge, most immunogenicity studies with EV-VLPs have also included adjuvants [12,13,14,15,16,17,18,49,50,51]. Adjuvants improve the quality, magnitude, and duration of the immune response, and generally, it is thought that vaccines composed of VLPs alone may not sufficiently trigger the innate immunity response to the level required for optimal stimulation of the adaptive immune system [48]. However, since we observed that nonadjuvanted CVB1-VLP produced a clear neutralizing immune reaction in our previous study [19], we wanted to explore the immunogenicity of CVB3-VLPs in C57BL/6J mice without the use of adjuvants. We hypothesized that VLPs should be able to induce robust immune responses, since the VLP structures present a repetitive arrangement of antigens [52], making them immunogenic without the need for immunomodulatory substances. CVB infections induce rapid and strong neutralizing antibody responses in mice [33,53] and humans [54] and the protective efficacy of vaccines correlates well with the magnitude of the antibody response [8,13,27,33,55]. In our previous studies with inactivated CVB1 whole-virus vaccines [55] and with CVB1-VLP vaccine [19] high total IgG and neutralizing antibody responses were induced in C57BL/6J male mice with non-adjuvanted vaccines and the responses were primarily Th2-type (IgG1) oriented. Here, the immune response of CVB3-VLP was like the previously measured immune response of inactivated whole-virus vaccines [55] and CVB1-VLP vaccine [19] in C57BL/6J male mice demonstrating the immunogenic potential of non-adjuvanted CVB3-VLP. In our previous study with CVB3-VLP, the immunogenicity of the VLP was studied in BALB/c mice in the presence of Freund’s adjuvant [13]. The total IgG and neutralizing antibody responses in the previous study [13] were slightly higher than the antibody responses obtained here without adjuvant, but it is important to note that the previous and the current experiment represent independent studies and the results obtained from different mice strains should not be compared quantitatively to each other. The structural and immunogenic data presented here indicate the potential of this improved methodology to produce highly immunogenic VLP-vaccines in the future.
